# Comparison of volume and hemodynamic effects of crystalloid, hydroxyethyl starch, and albumin in patients undergoing major abdominal surgery: a prospective observational study

**DOI:** 10.1186/s12871-020-01051-5

**Published:** 2020-06-03

**Authors:** Daisuke Toyoda, Yuichi Maki, Yasumasa Sakamoto, Junki Kinoshita, Risa Abe, Yoshifumi Kotake

**Affiliations:** Department of Anesthesiology, Toho University Ohashi Medical Center, 2-22-36 Ohashi, Meguro, Tokyo, 153-8515 Japan

**Keywords:** Goal-directed fluid management, General surgery, Hydroxyethyl starch, Albumin

## Abstract

**Background:**

The volume effect of iso-oncotic colloid is supposedly larger than crystalloid, but such differences are dependent on clinical context. The purpose of this single center observational study was to compare the volume and hemodynamic effects of crystalloid solution and colloid solution during surgical manipulation in patients undergoing major abdominal surgery.

**Methods:**

Subjects undergoing abdominal surgery for malignancies with intraoperative goal-directed fluid management were enrolled in this observational study. Fluid challenges consisted with 250 ml of either bicarbonate Ringer solution, 6% hydroxyethyl starch or 5% albumin were provided to maintain optimal stroke volume index. Hematocrit derived-plasma volume and colloid osmotic pressure was determined immediately before and 30 min after the fluid challenge. Data were expressed as median (IQR) and statistically compared with Kruskal-Wallis test.

**Results:**

One hundred thirty-nine fluid challenges in 65 patients were analyzed. Bicarbonate Ringer solution, 6% hydroxyethyl starch and 5% albumin were administered in 42, 49 and 48 instances, respectively. Plasma volume increased 7.3 (3.6–10.0) % and 6.3 (1.4–8.8) % 30 min after the fluid challenge with 6% hydroxyethyl starch and 5% albumin and these values are significantly larger than the value with bicarbonate Ringer solution (1.0 (− 2.7–2.3) %) Colloid osmotic pressure increased 0.6 (0.2–1.2) mmHg after the fluid challenge with 6% hydroxyethyl starch and 0.7(0.2–1.3) mmHg with 5% albumin but decreased 0.6 (0.2–1.2) mmHg after the fluid challenge with bicarbonate Ringer solution. The area under the curve of stroke volume index after fluid challenge was significantly larger after 6% hydroxyethyl starch or 5% albumin compared to bicarbonate Ringer solution.

**Conclusions:**

Fluid challenge with 6% hydroxyethyl starch and 5% albumin showed significantly larger volume and hemodynamic effects compared to bicarbonate Ringer solution during gastrointestinal surgery.

**Trial registration:**

UMIN Clinical Trial Registry UMIN000017964. Registered July 01, 2015.

## Background

Recent investigations have demonstrated the benefits of intraoperative goal-directed fluid management [1]. Although the typical protocol recommends rapid administration of rapidly degradable hydroxyethyl starch (HES) solution in order to optimize stroke volume [2–7], crystalloid and iso-oncotic albumin (Alb) have also been used for this purpose [8–10]. Traditionally, the distribution of fluid is thought to be dictated by the Starling principle. In this paradigm, iso-oncotic fluid, such as 6% HES and 5% Alb, remain in the vasculature, whereas isotonic, non-oncotic fluids are equally distributed throughout the entire extracellular space. Thus, iso-oncotic fluids should demonstrate a 3- to 4-times larger volume effect than crystalloid. Although this hypothesis is supported by the results of a study of healthy volunteers [11], clinical studies have repeatedly demonstrated that the difference in volume effect between colloid and crystalloid is much smaller than anticipated. Most available evidence suggests that the volume effects of HES and Alb are about 1.5 times larger than the volume effect of crystalloid [12]; however, only a few reports have directly compared volume and hemodynamic effects of crystalloid, HES, and Alb [10, 13]. Furthermore, the volume effect of an intravenous solution is typically assessed in surgical patients before or after intraperitoneal manipulation. Because volume effects are considered context-sensitive, data collect during periods of surgical stress and inflammation would be more clinically relevant than data collect from healthy volunteers or from surgical patients before or after surgery.

The purpose of this study was to compare the volume and hemodynamic effects of crystalloid, HES and Alb during intraoperative goal-directed fluid management in patients undergoing major abdominal surgery.

## Methods

This prospective observational study was approved by the Ethics Committee of Toho University Ohashi Medical Center (approval no. 14–13, approved on Feb. 10, 2014) and written informed consent was obtained from all subjects participating in this study. This study was registered prior to patient enrollment at UMIN Clinical Trial Registry (www.umin.ac.jp/ctr; UMIN000017964). During 18-month study period, we enrolled patients scheduled to undergo major, elective abdominal surgery for gastrointestinal, gynecological, or urological malignancies at Toho University Ohashi Medical Center. Patients were excluded from the study if they were younger than 20 years, were pregnant, had an arrhythmia, or underwent a laparoscopic procedure.

**Fig. 1:**
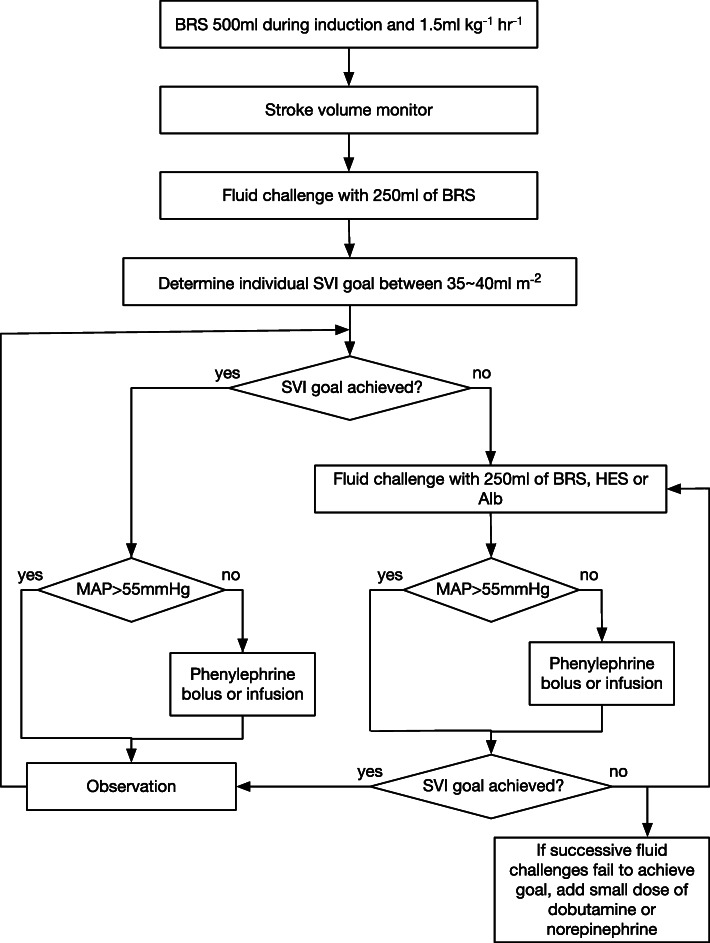
Protocol of goal-directed fluid management used in this study. SVI = stroke volume index; MAP = mean arterial pressure; BRS = bicarbonate Ringer’s solution; HES = 6% hydroxyethyl starch 130/0.4; Alb = 5% albumin

Prior to anesthesia induction, all study patients received an epidural catheter at mid to low thoracic level. General anesthesia was induced with combination of propofol, rocuronium, fentanyl and maintained with sevoflurane and remifentanil combined with intermittent rocuronium administration. Initially, 6 to 8 ml of 0.375% of ropivacaine was intermittently administered via epidural catheter. The timing and the dose of supplemental epidural ropivacaine were at the discretion of the attending anesthesiologists. Postoperatively, 0.2% ropivacaine supplemented with fentanyl was continuously administered via epidural catheter with elastometric pump. Patients were mechanically ventilated with a fixed tidal volume of 8 ml kg^− 1^ of predicted body weight and a positive end-expiratory pressure of 5 cmH_2_O. The respiratory rate was adjusted to maintain an end-tidal carbon dioxide level between 3.5 and 4.5 kPa. Bicarbonate Ringer’s solution (BRS; Bicanate, Ohtsuka Pharmaceutical Factory, Tokushima, Japan) [14] was administered at a rate of 1.5 ml kg^− 1^ h^− 1^. Either the left or right radial artery was cannulated with a 22-gauge Teflon catheter (Introcan Safety; BBraun, Melsungen, Germany), and stroke volume was continuously measured by non-calibrated arterial pulse contour analysis (FloTrac/Vigileo, ver. 3.04; Edwards Lifesciences, Irvine, CA). Air bubbles were removed from the line and the arterial catheter was carefully fixed at the wrist to prevent arterial waveform distortion. A central venous catheter was inserted when clinically necessary. The protocol of goal-directed fluid management used in this study was a modification of our previously reported protocol [15] and is summarized in Fig. 1. Briefly, the current protocol aimed to achieve an individualized stroke volume index (SVI) target by repeated fluid challenge [16]. Basically, the SVI > 35 ml m^− 2^ was used to the target of intraoperative hemodynamic target but target SVI up to 40 ml m^− 2^ is allowed dependent on the attending anesthesiologist discretion. Fluid challenges consisted of 250 ml of either BRS, 6% saline-based hydroxyethyl starch 130/0.4 (Voluven; Fresenius-Kabi, Bad Homburg, Germany, herein HES), or 5% albumin (CSL Behring, King of Prussia, PA, herein Alb). Each fluid challenge consisted of rapid injection of 250 ml fluid using a 50-ml syringe [5, 17] and a typical fluid challenge was finished in less than 5 min. BRS and HES were alternately used for the fluid challenge during the majority of the intraoperative period. In this study, Alb was selectively used during the late phase of surgery in long cases and in cases with significant blood loss. Additionally, phenylephrine was administered to maintain a mean arterial pressure greater than 55 mmHg. If the SVI goal could not be achieved with repeated fluid challenges, a small dose of either dobutamine or norepinephrine was administered. Other intraoperative care was at the discretion of the attending anesthesiologist.

Immediately before each fluid challenge, heparinized arterial blood was collected to determine the hematocrit (Hct) and plasma colloid osmotic pressure (COP). Hct was measured using a standard blood gas analyzer (Cobas b221; Roche Diagnostics, Basel, Switzerland). After centrifugation, plasma COP was measured using a colloid osmometer (Model 4420; Wescor, UK) with a semi-permeable membrane with a 30-kDa cutoff (SS-030). COP analysis was repeated 30 min after the start of each fluid challenge. This interval was based on our previous study wherein we found that the intraoperative volume effect of crystalloid disappears after about 30 min [15]. This interval also corresponds with previous volume kinetic studies that assessed the volume effect 30 min after the fluid infusion.

### Data analysis

In order to eliminate the confounding effects of blood loss and changes in vascular capacitance on the evaluation of volume effect, fluid challenges were excluded if they were concomitant with measurable blood loss, within 2 h after an epidural bolus injection of local anesthetic, vascular clamping/declamping, bolus administration or dose changes in the continuous infusion of vasoactive drugs, and if an additional fluid challenge was required during the 30-min observation period. The change of plasma volume caused by each fluid challenge was evaluated using the following formula [18]:

$$100\times \left(\mathrm{pre}\hbox{-} \mathrm{challenge}\ \mathrm{Hct}/\mathrm{post}\hbox{-} \mathrm{challenge}\ \mathrm{Hct}\hbox{-} 1\right)/\left(1\hbox{-} \mathrm{pre}\hbox{-} \mathrm{challenge}\ \mathrm{Hct}\right).$$

The hemodynamic effects of each fluid challenge were evaluated as follows. First, the trend of SVI was examined offline and the peak and duration of the SVI change caused by each fluid challenge were determined by two authors who were not involved in the intraoperative management (DT and YK). Then, the time to peak SVI, maximal SVI change, area under the curve of SVI change above baseline, and mean arterial pressure (MAP) change at the time of maximal SVI change were determined and compared.

Statistical analysis was performed with customized version of R software, ver. 3.4.4 (Foundation for Statistical Computing, Vienna, Austria) [19] and Prism software, ver. 7 (Graphpad Software Inc., La Jolla, CA). The normality of distribution was examined with the Shapiro-Wilk test. Data are expressed as either mean ± SD or median (interquartile range (IQR)), according to the distribution. Differences in the volume and hemodynamic effects between the three fluids were examined with either one-way analysis of variance or the Kruskal-Wallis test, depending on the data distribution. Either the Turkey test or Dunn’s test was used for post hoc comparisons of BRS, HES, and Alb. *P* < 0.05 was considered statistically significant. In this study, we hypothesized that the volume effect of colloid would be 50% larger than the volume effect of crystalloid. Based on this hypothesis, we estimated that 40 measurements for each fluid type were needed to achieve a beta error less than 0.8 and an alpha error of 0.05.

## Results

**Fig. 2:**
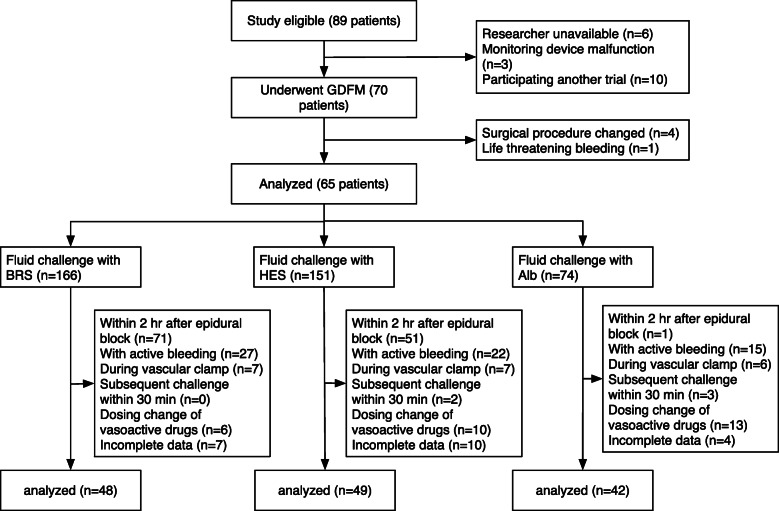
Flow of data analysis. GDFM = goal-directed fluid management; BRS = bicarbonate Ringer’s solution; HES = 6% hydroxyethyl starch 130/0.4; Alb = 5% albumin

The flow of patients and data analysis is summarized in Fig. 2. Of the 89 patients who met the inclusion criteria, 65 patients were included in the analysis. Most patients underwent midline laparotomy while the patients underwent hepatectomy received both midline laparotomy and subcostal incision. Patient demographics and surgical data are summarized in Table 1, respectively. A total of 391 fluid challenges were performed; however, 252 fluid challenges were excluded from the analysis based on the predetermined exclusion criteria. Finally, 48 fluid challenges with BRS, 49 fluid challenges with HES, and 42 fluid challenges with Alb were included in the analysis (Fig. 2). Since fluid challenges administered within 2 h of epidural administration of local anesthetics were excluded, the mean interval between incision and each analyzed fluid challenge was 210 ± 108 min for BRS, 209 ± 93 min for HES, and 309 ± 111 min for Alb. Most of these fluid challenges occurred relatively late in the surgery, especially during periods of significant surgical stress due to intraperitoneal manipulation.

**Table 1:** Perioperative data

Age (years)	67 ± 12
Sex (male/female)	40/25
Height (cm)	161 ± 10
Weight (kg)	59 ± 12
BMI (kg/m^2^)	22.5 ± 3.7
ASA PS (1/2/3/4)	9/36/19/1
Surgery type	
Upper gastrointestinal / Hepatobiliary / Colorectal / Gynecological / Urological	8/40/2/8/7
Duration of anesthesia (min)^a^	552 (413–711)
Intraoperative fluid administration (ml/kg/hr)	6.7 ± 1.7
Amount of perioperative crystalloid (ml)	2250 (1850–2550)
Amount of perioperative HES (ml)	500 (500–750)
Amount of HES as a percentage of total perioperative fluid	17 ± 6
Amount of intraoperative 5% albumin (ml) (*n* = 54)	250 (250–500)
Amount of 5% albumin as a percentage of total perioperative fluid (*n* = 54)	13 ± 5
Number of patients who received packed red blood cells/fresh frozen plasma/platelets	15/8/1
Estimated blood loss (ml)	390 (210–760)
Urine output (ml)	300 (160–430)

Data are expressed as number or mean ± standard deviation

*BMI* body mass index; *ASA PS* American Society of Anesthesiologists physical status

*HES* 6% hydroxyethyl starch 130/0.4

^a^: Duration of anesthesia is defined by Japanese regulatory agent as the duration when oxygen was administered via anesthetic circuit

**Fig. 3:**
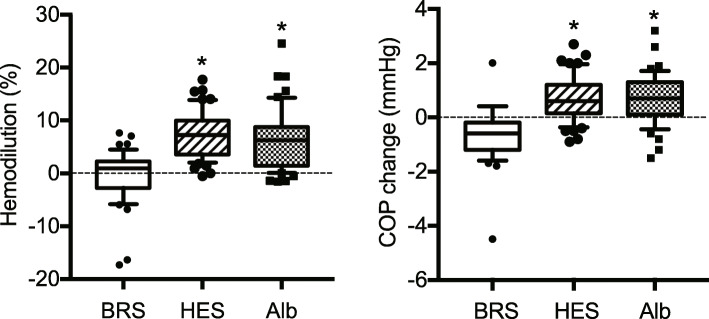
Changes in plasma volume and colloid osmotic pressure after fluid challenge. Box and whiskers represent median, interquartile range, and 10–90% range, respectively. *p < 0.05 vs. bicarbonate Ringer’s solution by Dunn’s post hoc test. BRS = bicarbonate Ringer’s solution; HES = 6% hydroxyethyl starch 130/0.4; Alb = 5% albumin

The median increase in plasma volume 30 min after fluid challenge was 7.3% (IQR, 3.6 to 10.0%) with HES and 6.3% (IQR, 1.4 to 8.8%) with Alb. Conversely, the median increase in plasma after fluid challenge with BRS was only 1.0% (IQR, ˗2.7 to 2.3%). Thus, the volume effects 30 min after fluid challenge with HES and Alb were significantly greater than with BRS; however, there was no significant difference between the volume effects of HES and Alb (Fig. 3, left panel). Similarly, COP increased by 0.6 mmHg (IQR, 0.2 to 1.2 mmHg) after fluid challenge with HES and 0.7 mmHg (IQR, 0.2 to 1.3 mmHg) after fluid challenge with Alb; however, COP decreased by 0.6 mmHg (IQR, 0.2 to 1.2 mmHg) after fluid challenge with BRS. Thus, COP changes after fluid challenges with HES or Alb were significantly greater than after fluid challenge with BRS (Fig. 3, right panel). Notably, there was no significant correlation between the COP change and volume effect after fluid challenge with any of the three study fluids (R^2^ between the volume effect after BRS, HES and Alb was 0.09, 0.17 and 0.02, respectively).

**Fig. 4:**
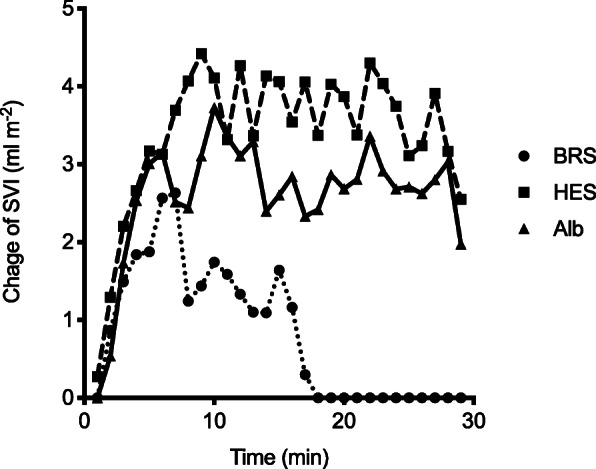
Time course of increase in stroke volume index after fluid challenge. For clarity, only median values of the change in stroke volume index are shown. SVI = stroke volume index; BRS = bicarbonate Ringer’s solution; HES = 6% hydroxyethyl starch 130/0.4; Alb = 5% albumin

The course of SVI after fluid challenge with each fluid is demonstrated in Fig. 4. The time from the start of the fluid challenge to the peak SVI was similar between the three study fluids. The median SVI increase was 5 ml m^− 2^ (IQR, 3 to 8 ml m^− 2^) with BRS, 8 ml m^− 2^ (IQR, 5 to 12 ml m^− 2^) with HES, and 5 ml m^− 2^ (IQR, 3 to 8 ml m^− 2^) with Alb. The maximal SVI was significantly higher after fluid challenge with HES than with BRS or Alb (*p* < 0.0001). After the fluid challenges, the median area under the curve of SVI was 26 ml m^− 2^ (IQR, 11 to 63 ml m^− 2^) with BRS, 107 ml m^− 2^ (IQR, 53 to 159 ml m^− 2^) with HES, and 80 ml m^− 2^ (IQR, 33 to 138 ml m^− 2^) with Alb; notably, the SVI was significantly higher after fluid challenge with HES or Alb than with BRS (*p* < 0.0001). The median MAP increase at the time of peak SVI was 2.5 mmHg (IQR, 0 to 8 mmHg) with BRS, 6 mmHg (IQR, ˗1 to 11 mmHg) with HES, and 4 mmHg (IQR, 0 to 7.5 mmHg) with Alb; however, there were no significant differences in the MAP increase between the three study fluids.

## Discussion

In this study, we compared the volume and hemodynamic effects of fluid challenges with crystalloid, 6% HES 130/0.4, and 5% Alb during surgical manipulation in patients undergoing major abdominal surgery. We found greater hemodilution, as well as a larger increase in COP and SVI, after fluid challenges with HES and Alb than with crystalloid.

The current study has two distinct features. First, all fluid challenges were rapid (finishing in less than 30 min). A meta-analysis by Toscani et al. revealed that fluid challenges that finished in less than 30 min resulted in a higher proportion of responders compared with fluid challenges that took longer than 30 min [20]. Aya et al. recently found that a 4 ml kg^− 1^ bolus over 5 min was adequate to reliably discriminate fluid responders from non-responders in post-cardiac surgical patients [21]. Furthermore, Miller et al. recommended a fluid challenge consisting of 5 consecutive injections of 50 ml by syringe push for goal-directed fluid management, which is the method adopted in the present study [5]. Collectively, our protocol corresponds well with recent studies and likely represents the contemporary standard of care for intraoperative goal-directed fluid management. Second, the volume effects of each study fluid were evaluated during actual surgical stress. Volume effects of administered fluids are considered context-sensitive [22, 23], and surgical manipulation and inflammatory response both increase vascular permeability, resulting in a significant fluid shift from the intravascular to the extravascular space [24–26]. Thus, the results of this study may be more clinically relevant than studies of healthy volunteers or surgical patients before or after surgery.

Increases in plasma volume were sustained for at least 30 min after fluid challenge with HES or Alb, but not with BRS. In patients undergoing major abdominal surgery, intravascular volume may be continuously lost to the interstitial space due to surgical stress and inflammation, as well as to the environment through evaporative loss. These findings suggest that, despite these fluid shifts, a significant proportion of administered HES or Alb remains intravascular, whereas a significant amount of administered BRS is lost from the vasculature after 30 min. Joosten and the colleagues analyzed the data of existing trial and found that the initial hemodynamic change during fluid challenge is independent of the types of fluid [27]. They speculated that the lower number of boluses required to achieve hemodynamic target might be related to the longer intravascular persistence of the colloid solution. Our data support their speculation that the volume effect of crystalloid and colloid solutions becomes gradually different in the later phase after fluid challenge. There was a slight increase in COP 30 min after fluid challenge with HES or Alb; however, COP decreased 30 min after fluid challenge with BRS. Two clinical studies of healthy volunteers demonstrated slightly increased COP after colloid infusion [28, 29], and our data are basically in line with these previous reports. Therefore, we speculate that COP changes are at least partially due to the different volume effects of colloid and crystalloid.

Since fluid administration is generally guided by either subjective decision of the attending anesthesia providers or objective hemodynamic parameters, the difference in volume effects between crystalloid and colloid in many clinical studies likely reflects differences in hemodynamic effects. Unfortunately, reports with detailed hemodynamic profiles after fluid challenges are rare. Aya et al. reported that cardiac output peaked 1 min after fluid challenge with 250 ml crystalloid in postoperative ICU patients and the effect was sustained for about 10 min after the completion of the fluid challenge [17]. Gandos et al. reported that the area under the curve of cardiac index was significantly higher after fluid challenges with HES or Alb than with crystalloid [13]. Our findings basically agree with these previous reports; however, they also provide interesting insights about the hemodynamic effects of fluid challenges. Fluid challenge with HES resulted in a higher peak SVI than fluid challenge with Alb; however, the area under the curve of SVI was not statistically different between fluid challenges with HES or Alb. Collectively, our data confirm that colloid, such as HES and Alb, generate larger hemodynamic effects than crystalloid. In addition, our data support the observed differences in volume effects between colloid and crystalloid [12, 30].

We believe that the results of this study have clinical implications. HES was not associated with the improvement of the majority of the outcomes despite the lower fluid requirement in the recent large-scale randomized trial. Instead, the authors found increased incidence of low-stage AKI and concluded that use of HES is not supported [31]. However, one of the co-authors of this manuscript found that intraoperative HES use is associated with low stage AKI but is not associated with advanced stages of AKI, the use of renal replacement study or increased mortality in the large retrospective study [32]. Furthermore, slightly positive fluid balance at the end of surgery is recommended based on the results of large-scale trial [33, 34]. Such fluid balance may be achieved without colloid in cases with 2 to 3 h of duration, we assume achieving such balance without colloid is difficult during more invasive, extensive surgeries. In this regard, we agree with the recent editorial comment which supports the use of HES in the contemporary surgical environment [35].

This study has several limitations. First, the context of each fluid challenge significantly affects data interpretation. We tried to minimize the influence of factors such as blood loss, changes in vascular compliance (epidural blockade and vasopressor use), and surgical manipulation. Nevertheless, such adjustments still remain subjective and cannot preclude the presence of confounding factors. The robust results found in this study suggest that the volume effects and subsequent hemodynamic effects of the study fluids are real. Second, COP was determined using a semipermeable filter with a cut-off value of 30 kDa. The renal excretion threshold is around 50 kDa [36]; therefore, molecules with a molecular weight between 30 and 50 kDa contribute to the COP value measured by the osmometer but are not osmotically active in vivo. Because this issue is particularly relevant to HES, this study may have overestimated the effect of HES on COP. Third, we did not fully account for the interaction between HES and BRS. Hahn et al. reported that the volume effect of acetate Ringer’s solution was modified by the preceding administration of HES [37]. Since all patients in the present study received both HES and BRS, the results may be affected by this interaction; however, we believe that the current protocol represents a realistic balance of crystalloid and colloid, which can maximize the benefits of goal-directed fluid management and prevent dose-dependent side effects of HES, especially in long cases. Fourth, this study included a significant number of elderly patients and the subjects with multiple comorbidities such as with ASA PS 3, which may limit the generalizability of these results. Fifth, the fluctuation of RBC size and Hct during long surgical procedure may affect the accuracy of plasma volume calculation. Sixth, several formulas other than the one used in this study are used in the literature and it is not clear whether the current formula is most adequate in this setting or not. Despite these limitations, the current study demonstrates a significant difference in the volume and hemodynamic effects of crystalloid and colloid during surgical manipulation under general anesthesia.

In conclusion, this study showed that the increase in plasma volume after rapid injection of crystalloid during major abdominal surgery was almost completely lost after 30 min. Conversely, rapid injection of both HES and Alb resulted in significantly greater increases in plasma volume and COP compared with BRS. Moreover, increases in plasma volume were accompanied by concomitant increases in stroke volume. These results correspond well with the results of other recent studies and confirm that colloid can reduce the total fluid input during goal-directed fluid management.

## Data Availability

The datasets used and analyzed during the current study are available from the corresponding author on reasonable request.

## Abbreviations

HES: Hydroxyethyl starch
Alb: Albumin
BRS: Bicarbonate Ringer solution
COP: Colloid osmotic pressure
SVI: Stroke volume index
Hct: Hematocrit
